# Supplementary material for: Are carbon emissions trading and green financial instruments synergistic? -Comprehensive quantitative research based on content analysis
Source: PLoS One. 2024 Mar 7;19(3):e0298601. doi: 10.1371/journal.pone.0298601 (PMC10919601; doi:10.1371/journal.pone.0298601)
Supplement: S1 Data — (ZIP) [file pone.0298601.s001.zip › Data/地级市Z_F工作报告.zip!/║■─╧/╧μ╠╢╩╨/└┤╫╘╣1⁄2╓┌║┼í╛┬φ┐╦╩2╛▌═°í┐_2015.docx]

各位代表：

现在，我代表市人民政府向大会报告工作，请予审查，并请各位政协委员和其他列席人员提出意见。

一、2014年工作回顾

过去一年，在省委、省政府和市委的坚强领导下，我们紧密团结和依靠全市人民，沉着应对经济下行压力，落实稳中求进系列措施，实现地区生产总值1570.6亿元，同比增长10.7%；固定资产投资1503.4亿元，增长23.7%；规模工业增加值870.1亿元，增长11%；财政总收入159.4亿元，增长10.1%；社会消费品零售总额450亿元，增长13%；城乡居民人均收入分别为27068元、14092元，增长9.1%、11.2%。省定节能减排任务全面完成。尽管部分指标离预期目标有些差距，但经济社会总体保持平稳健康发展。我们着力做了以下工作：

着力稳增长，经济运行总体平稳。稳企业。出台和落实促进企业发展的系列政策措施，实行重点企业点对点帮扶，设立中小微企业过桥专项资金，加强银企对接等，着力帮助企业解决融资难题，信贷规模保持适度增长，全年新增银行贷款168.6亿元。企业发展逐步趋稳，规模工业总产值达2937.8亿元，增长11.2%。稳投资。实施重点项目240个，完成投资突破800亿元。产业投资占固定资产投资比重达41.7%。稳消费。积极培育消费热点，优化消费结构，居民消费水平不断提升。住房消费基本稳定，汽车等大宗消费持续增长，旅游休闲消费趋旺。新增水府旅游区、昭山风景名胜区、盘龙大观园3个国家4A级旅游景区。全年接待游客突破3000万人次。稳重点区域。湘潭经开区、湘潭高新区规模工业总产值分别增长23.2%、18%。昭山示范区生态休闲、文化创意产业发展基础不断夯实。湘潭县、韶山市推进全省特色县域经济重点县建设进展良好。湘潭县启动“一区多园”模式发展乡镇工业。湘乡市园区发展、城市建设步伐加快。3个县(市)全面小康实现程度提升5个百分点以上。

着力调结构，发展格局逐步向好。积极引进和培育新产业、新业态，泰富重装产值突破百亿大关，吉利汽车首个新能源战略项目落户湘潭，湘潭电化新基地正式投产。威胜电气产业园、上海大众汽车配件产业园等重大工业项目，华拓数码、中国网库等高端生产性服务业项目，万达广场、中国(中部)国际商贸城等大型城市综合体项目进展顺利。坚持以创新促转型，风力发电装备制造获批省级创新型产业集群试点，湘电2兆瓦级超低风速直驱式智能型风力电机组等2项科技成果入选全省产业技术创新10大标志性成果，工矿电传动车辆国检中心投入运行，市产业创新研究院、湘潭大学科技园挂牌运行。完成高新技术产业总产值1576.6亿元，增长23.3%。新增中国驰名商标2件。加快开放平台建设，湘潭综合保税区顺利通过省预验收，韶山出入境检验检疫局正式运行。完成进出口总额23.7亿美元。实际利用外资8.3亿美元，增长18.8%。着力优化农业结构，粮食等主宗农产品保持稳产增产，农业产业化总产值达630亿元、增长18%；规模以上农产品加工业产值与农业产值比由2.2：1提高到2.4：1，处于全省领先水平。三次产业结构由上年8.4：58.9：32.7调整为8.4：57.3：34.3。非公经济占地区生产总值比重达66.7%，提高3.8个百分点。

着力推改革，创新活力不断增强。推进行政管理体制改革，取消、调整、下放行政审批权限172项，市本级行政审批项目较上年精简20.7%。完成城区食品药品监管体制改革。市直事业单位完成分类。预算绩效管理覆盖市本级一级预算单位，经验在全国推介。42家市属国有企业完成改制。驻潭央企省企分离移交办社会职能工作稳步推进。着力推进商事制度改革，新设企业增长50%。开展PPP模式试点，第一批42个项目向社会资本开放，湘潭经开区污水处理一期工程被列为全国30个示范项目之一。第一个代建制项目市体校交付使用。推进社会领域改革。城乡居民养老保险实现并轨。在城市区逐步实行农村低保、“五保”城乡一体化。在湘乡市启动县级公立医院改革试点。完成行政村调整合并，全市行政村数量减少299个。开展农村土地确权登记颁证和农村土地承包经营权抵押贷款试点。耕地流转70.1万亩，流转率达40.3%。农民合作社、家庭农场分别达到1100家和210家。积极稳妥推进价格改革，进一步加强市场价格监管，物价总水平保持稳定。城市居民用水实行阶梯水价 侯卫东官场笔记。排污权有偿使用和交易范围扩大到全市所有工业企业排污单位。

着力抓建设，城乡面貌持续改观。重大基础设施建设顺利推进。沪昆高铁湘潭站、韶山站投入运行，湘潭迈入“高铁时代”。长韶娄高速公路正式通车，域内高速公路达到7条。长株潭城际铁路湘潭段加快建设，九华大道、芙蓉大道二期、天易路二期等城际道路建设进展良好。湘乡-壶天、锰矿-湘潭等干线公路快速推进。实施北二环提质改造。东二环及五大桥、高新路等竣工通车。开工建设G320绕城线城际快车道一期(含昭华大桥)和白云路。湘江风光带新建成通车14.7公里。编制绿地系统、景观风貌等23项专项规划。开展城市绿荫行动，启动城区绿道建设，湖湘公园二期、木鱼湖公园一期基本建成。国家园林城市顺利通过复查。改造老旧社区5个。河西平战结合人防工程全面建成。以创建全国文明城市为抓手，加大城管考评力度，集中开展渣土运输等“五项”整治，持续推进城市净化、美化、亮化工程。规范城区338条道路名称。调整优化公交线路55条，开通全省首条城乡客运一体化示范公交线路。加强和规范出租车行业管理，依法调整出租车运价和特许经营，依法打击非法营运行为。治理超限超载，“双超”率由40%下降到3.8%左右。小城镇和美丽乡村建设进展较好。启动建设花石、棋梓、银田3个特色镇。梅林桥城乡统筹示范片、姜畲现代农业示范区建设顺利推进。农村环境卫生整治“三年行动计划”全面完成。韶山市启动全域城乡环境卫生综合整治。新建农村公路587公里，改造危桥62座。完成“三边”造林1.5万亩。完成水利建设投入12.8亿元，创历史新高。建设“五小”水利工程3.2万处，解决21万人饮水安全问题。3个县(市)和雨湖区成为全国小型农田水利建设重点县。实施鹤岭地区和杨梅洲供水工程，解决3万余人供水问题。推进锰矿地区治理和建设，关停重污染涉锰企业28家，废渣废水治理工程即将竣工。大力推进落后小煤矿关闭退出，湘潭县成功关闭煤矿5家。湘江保护和治理“一号重点工程”实现重大突破。竹埠港28家重化工企业全部关停，创造了在全省可推广的经验。河西、河东污水处理厂改扩建工程进展顺利。湘江湘潭段全面禁止采砂。超额完成湘江干流两岸规模养殖退出省定年度任务。加强大气污染防治，空气优良率提高14.2个百分点。

着力惠民生，保持社会和谐稳定。民生支出占公共财政支出比重提高到63%。省、市为民办实事任务全面完成。新增城镇就业6.4万人，新增农村劳动力转移就业1.6万人。

社会保障水平全省领先，职工医保统筹基金和大病医疗互助的最高支付限额分别由6万元、18万元提高到9万元、25万元，失业保险金发放标准调整为每月1012元。全面实施基本药物制度，新农合惠及农村居民194.9万人，参合率达98.2%。在全省率先启动社会保障卡应用工作。为2.5万余名重度残疾人发放生活补助和护理补贴近3000万元。建成保障性住房和棚户区改造住房15735套，改造农村危房3050户，改扩建农村敬老院12所。强化住房公积金归集扩面，提高了职工住房消费支持能力。促进城乡教育均衡发展。建成农村义务教育合格学校92所、公办幼儿园9所。建成农村教师周转房372套。韶山市、雨湖区、岳塘区全面完成完全小学以上义务教育合格学校建设。城区新增义务教育学位6876个，大班额问题基本化解。高中学业水平考试合格率居全省第一。市工贸中专通过国家示范校验收。启动实施“单独二孩”政策。人口计生工作连续6年保持全省模范。完成140个村卫生室建设。雨湖区成功创建为国家慢性病综合防控示范区。在全省率先开展“全民健身示范城市”创建。成功举办全省全民健身节。组织开展“欢乐潇湘·幸福湘潭”群众文艺汇演百余场次。大型实景演出《中国出了个毛泽东》正式公演。电视连续剧《彭德怀元帅》开拍。《东山学堂》获省“五个一工程奖”。严厉打击食品药品违法行为，有效保障了食品药品安全。开展百日安全大排查大整治行动，安全生产事故和事故死亡人数实现双下降。市人民来访接待中心正式启用。一批重大疑难信访积案得到有效化解。全面强化立体治安防控，社会治安秩序明显好转。国防动员和后备力量建设扎实推进，全民国防意识明显加强。外事工作获中国国际友好城市交流合作奖。妇女儿童、青少年、老龄、民族宗教、档案、供销、移民、气象、水文等工作取得新进展。中央、省驻潭单位为促进湘潭经济社会发展作出了积极贡献。

着力促规范，政府效能进一步提升。深入开展党的群众路线教育实践活动，坚决贯彻落实中央关于作风建设的各项规定，聚焦“四风”问题，依法规范行政行为，提高行政效能。实施行政审批制度改革“1+3”清单管理。市政务服务中心正式运行，首批343项审批服务事项进入中心。依法清理税费优惠政策，规范与行政职权关联的中介机构、行业协会收费行为。推行建设项目“一表制”收费管理。对市本级政府投资项目全面推行预算、结算、决算“三审制”。规范土地管理，发展建设用地得到较好保障，耕地得到较好保护，土地市场供大于求调控初见成效。加强行政决策合法性审查，健全政府合同审查管理机制，防范行政决策风险。设立市本级政府偿债准备金，制定债务管理办法，着力防范和化解政府性债务风险。严格财政支出管理，“三公”经费在上年决算数基础上压减20%。完善政府投融资公司“三重一大”决策制度。在湘潭县开展委托乡镇执法试点。自觉接受人大、政协和社会各界监督，高度重视人大代表建议和政协提案办理工作，全年建议提案办结率为100%。积极采纳代表委员意见建议，提高行政决策水平和行政效率。强化正风肃纪，严肃查处政府工作人员违纪违规和贪腐行为，形成了强大的震慑力。

各位代表，尽管去年工作压力很大，但各级各部门各单位在力争实现全年目标任务的同时，自觉负重奋进，积极创先争优，系列重大工作跻身国省平台。长株潭国家自主创新示范区获国务院批准，湘潭获批创建全国首家红色旅游融合发展示范区、国家知识产权示范城市、全国质量强市示范城市、全国养老服务业综合改革试点城市、全国社会保障卡综合应用示范城市，成功申报创建国家现代农业示范区，先进矿山装备集群成为国家级创新型产业集群试点，韶山灌区获批国家水利风景区，等等。这些，都为进一步的发展打下了坚实基础。

回顾过去一年，成绩来之不易，经验弥足珍贵。这是在中共湘潭市委的正确领导下，全市上下深入贯彻落实中央和省委省政府系列决策部署、奋力拼搏的结果。在此，我代表市人民政府，向辛勤工作在各行各业的广大干部群众，向人大代表、政协委员，向各民主党派、工商联、人民团体、无党派人士和离退休老同志，向驻潭人民解放军、武警官兵和政法干警，向所有关心、支持湘潭改革发展的各界人士，表示衷心的感谢!

总结成绩的同时，我们感到困难和问题仍然严峻。受宏观经济形势和我市经济结构动力不足的双重影响，部分经济发展指标没有达到年初预定的奋斗目标；发展方式总体仍然粗放，新产业培育还不够，传统产业发展困难较大，转型升级任务艰巨；城市宜居水平离群众的愿望还有差距，城市设计、功能配套、管理精细化等亟需加强；农村基础设施仍相对薄弱，环境卫生和公共服务有待进一步改善；改革任务繁重，制约发展的思想观念、体制机制障碍等还不同程度存在；作风建设还需加强，一些部门服务意识仍然不强，部分干部仍存在求稳怕乱、缺少担当、劲头不足等问题。对此，我们必须强化措施，切实加以解决。二、2015年工作目标、任务和重点

2015年是全面深化改革的关键之年，也是全面完成“十二五”规划的收官之年。国际环境仍然复杂多变，世界经济复苏缓慢、曲折向好。我国经济已进入增速换挡、结构调优、动力转换的新常态。国家实施“史上最严”《环保法》，对发展既是机遇，也是严峻考验。湘潭经济增长虽有下行压力，但长期向好的趋势没有改变。我市经济总量已有较大规模，产业结构在不断优化，改革效应正逐步释放，随着国家实施长江经济带战略、长株潭获批自主创新示范区、沪昆高铁通车、综合保税区封关运行等，还将迎来更多机遇，完全有基础、有条件保持经济平稳健康发展。我们必须坚定信心，抢抓机遇，实现新常态下的新作为。

政府工作总体要求是：认真贯彻落实党的十八大和十八届三中、四中全会精神，按照中央、省委、市委经济工作会议要求，坚持稳中求进工作总基调，以提高发展质量和效益为中心，深入推进“四化两型”、“三量齐升”、“两个率先”，更加突出转方式调结构，更加突出改革攻坚，更加突出创新驱动，强化风险防控，加强民生保障，保持经济社会平稳健康发展，为提前全面建成小康社会、迈向基本实现现代化奠定坚实基础。

主要预期目标是：地区生产总值增长9%左右；固定资产投资增长20% 天火大道无弹窗；规模工业增加值增长9.5%以上；财政总收入增长9%以上；社会消费品零售总额增长12.5%；进出口总额增长10%；实际利用外资增长10%；城乡居民人均收入分别增长10%和11%；万元GDP综合能耗下降3.7%，全面完成“十二五”减排任务；人口自然增长率控制在6.5‰以内。重点抓好以下工作：

(一)坚持稳增长、调结构，优化发展格局。

推进项目建设，不断扩大有效投资。依靠优质项目促进结构调整，培育新的增长点和持续增长动力。全年安排重点项目300个左右，年投资800亿元左右。突出抓好泰富重工二期、中国(中部)国际商贸城等10大产业项目，杨梅洲大桥、湘江风光带等10大基础设施项目，竹埠港“退二进三”、河东第二污水处理厂等10大生态环保项目，保障性安居工程、农村饮水安全工程等10大社会民生项目。完善项目推进机制，促进项目顺利建设和竣工达效。集中力量抓好战略招商引资，瞄准世界500强、行业龙头和“补链强链”企业等，加强项目储备和前期工作，创新和完善招商引资方式，增强招商韧劲，全年引进市外资金275亿元以上，推动品新科技、清华同方等优质项目签约入驻。实施全市统一的招商引资政策，统筹项目引进和布局，提高招商效率。

注重企业培育，做强做优市场主体。高度重视国有大中型企业发展。优化环境，加强服务，促进驻潭央企、省企发展壮大。积极支持湘钢调整产品结构，实现销售收入400亿元以上；湘电加快风电产业转型升级，培育和发展海上风力发电产业，打造新能源装备制造领军企业；江南、江麓加大军民结合产品研发力度，打造军民融合型、质量效益型企业；江滨加快技术研发和创新，打造世界一流的汽车零部件供应商。推动市属国有企业加快战略性重组，以产业链为纽带，整合形成一批集团公司。支持非公经济发展。大力推动步步高集团等进一步做大做强。促进湘潭电化、华拓数码等一批成长性好的企业加快发展。鼓励中小型企业通过加强科技创新提升产品附加值，通过对接资本市场实行股份制改造，以技术和管理的双轮驱动带动转型升级。推动大众创业、万众创新，加快培育一批创新型、创业型、成长型小微企业，引导、扶持个体工商户升级为企业。完善中小微企业服务体系，认真落实支持企业发展的政策。加强融资性担保体系建设，探索风险分担机制，充分发挥政府全资融资性担保公司的引导作用，缓解中小微企业融资难题。严厉打击非法集资，积极稳妥开辟渠道引导民间投资。完善监测和预警机制，强化风险防控，促进企业规范稳健发展。

致力产业振兴，优化产业结构。加速推进新型工业化。以智能制造为主攻方向，积极引进和培育3D打印、工业机器人、集成电路等智能产业和先进装备制造、新能源汽车、电子信息、新材料、节能环保、生物医药等新兴产业。加快恒润高科整车生产基地、上海斐讯大数据中心等项目建设，力争泰富重工二期、威胜电气产业园、吉利新能源汽车、湘潭高新区企业加速器等项目竣工投产。推动信息化与工业化融合、制造业与服务业融合。加快推进机电、冶金、建材、食品等传统产业智能化、低碳化改造。积极推进住宅产业化。加快发展新兴服务业。大力发展移动互联网、电子商务、会展经济、研发设计、节能环保、信息消费、快递等业态。支持星宇电商物流等电商企业发展，推动传统企业向电商领域拓展，引进和培育电商代运营机构，做大电商平台，打造网商聚集区。抓住互联网生态圈发展机遇，逐步将岳塘经开区打造成长株潭供应链核心枢纽。以湖南一力物流公路港为龙头，逐步将雨湖工业集中区打造成区域性工业物流基地。改造提升商贸流通、住宿餐饮等业态，大力培育核心商圈、专业市场、市场群和特色街区。继续推进义乌小商品城建设。努力创建全国红色旅游融合发展示范区，完善以旅游交通、智慧旅游为重点的服务体系，促进旅游产业升级发展。大力发展文化产业，重点抓好窑湾历史文化街区、昭山文化创意产业园区、润泽东方文化产业城二期等项目建设。推进湘潭国家现代农业示范区建设。在稳定粮食播种面积和总产的基础上，以发展“两型”农业、都市农业、设施农业、高效农业等为重点，打造3家以上现代农业示范园，打造5个连片10万亩以上的优质稻高产示范片，建设10个千亩以上蔬菜产业园和2个万亩以上蔬菜示范片，高标准建设3个万亩油茶和2个万亩楠竹示范片。推进生猪标准化养殖，抓好沙子岭猪品牌开发，提升养殖品质和效益。引导工商资本发展高标准设施农业、规模化养殖等现代种养业。引导土地流转，促进适度规模经营。大力推进农业机械化，加强农村土地综合整治，提高农业综合生产能力。积极打造科技支农平台，加快湘潭国家农业科技园区建设。提高农业产业化水平，规模以上农产品加工企业总产值和增加值均增长15%左右。加强国省粮食仓储物流项目建设，抓好国省下达新增地方粮食储备任务。促进科技创新与产业紧密结合。以长株潭国家自主创新示范区建设为契机，以驻潭高校为智力支撑，以市产业创新研究院为平台，整合全市科教资源，加强人才队伍和项目建设，围绕产业链布局创新链，打通技术研发、产品开发、产业化生产等关键环节，加快培育一批新兴产业。引导企业加大科技投入，坚持自主研发和引进消化吸收再创新相结合，突破一批行业核心技术。加强产学研结合，支持高校和科研院所科技成果进园区、进企业。积极支持湘潭大学、湖南科技大学、湖南工程学院成为产学研结合的示范基地。立足湘潭军工技术优势，大力推进军民融合发展，重点发展工程机械、清洁能源和新能源装备、大型矿山装备、海工装备、智能装备等产品，努力把军民融合产业打造成湘潭具有规模优势的特色产业。

布局“一轴一带”经济走廊，积极融入长江经济带。抢抓国家重点实施长江经济带战略机遇，规划和布局“湘江高端服务创新产业轴”、“环线经济带”等“一轴一带”经济走廊，“一轴”重点发展高端服务业，“一带”重点发展工业和生产性服务业，形成错位发展、优势互补局面。“湘江高端服务创新产业轴”依托湘江黄金水道，对接长沙湘江新区，以湘江两岸为核心空间，布局滨江经济，重点发展金融、商务、现代物流、科技创新、文化创意、休闲旅游等高端服务业。近期重点完善规划，加强项目策划、引进和建设。切实加强对沿江建设的管控，在建高端服务业项目严格按规划实施，其他项目要优化调整，确保不破坏“创新产业轴”格局，为下一步布局产业赢取主动。“环线经济带”依托已建成的北二环、东二环，整合环线区域的产业、科教、区位等优势，加强互联互通建设，培育连接长、株、潭，贯串湘潭高新区、湘潭经开区、昭山示范区、天易示范区、岳塘经开区、雨湖工业集中区等园区以及多所高校和科研院所的环线经济带。依托“环线经济带”加强统筹，强化招商合作和产业配套，促进园区、产业之间协同、融合发展，避免产业同质同构。今年，抓紧“环线经济带”的规划、布局和建设，依托已有园区的产业和其他相关产业，完善产业链群，先期推进北二环、东二环区域产业发展；西二环、南二环区域按经济带要求进行规划控制，为未来发展预留空间。

提升各区域发展水平，优化多点支撑格局。加快园区转型发展。落实省政府创新创业园区发展“135”工程部署，推动园区“二次创业”，打造园区升级版。突出主导产业立园，加大全市园区发展统筹力度，进一步明确各园区定位，分类实施统一的政策和管理，促进园区错位、特色发展。昭山示范区、岳塘经开区大力发展现代服务业，其他园区突出工业主体地位，推进科技创新和转型升级，因地制宜发展各具特色的产业集群。全面跟进园区配套建设，积极发展匹配工业的生产性服务业，加快标准厂房建设，提升园区承载能力。大力创新园区管理体制和内部运行机制，提升园区服务企业的能力。推动县域经济特色发展。鼓励、支持县(市)加强产业规划，进一步优化功能分区。支持湘潭县以农副产品加工为主、湘乡市以输变电及电气设备为主，加快发展特色经济。支持韶山核心景区实行封闭管理，加快打造全省红色旅游集散中心和旅游目的地。大力发展县域园区，支持园区围绕主导产业，加强重大项目引进和建设，鼓励采取“一区多园”、“飞地经济”等模式，在有条件的地区设立经济区或乡镇园区。推行“园区+生产基地”模式，发挥工业对农业的带动作用。增强乡镇的经济功能，引导乡镇根据当地特色资源，做强特色产业，努力打造一批经济强镇。发挥综保区优势，壮大开放型经济。全力支持湘潭综保区建设，引进一批外向型企业，加快配套项目建设，确保年内封关运行，带动全市外向型经济发展。推动外贸转型升级，培育壮大一批外贸企业，引导企业打造外贸优势品牌，支持有条件的企业“走出去”，推动精品钢材、农副产品、特种机电等产品出口。大力发展服务外包产业，培育和引进服务外包企业20家以上。

(二)推动改革取得实质性进展，增强全社会发展活力。

推进经济体制改革。加快推进企业改革改制。巩固市属国有企业改制成果，加快推进“事改企”工作，稳妥解决企业改制历史遗留问题。积极配合央企省企分离办社会职能。努力发展混合所有制经济，支持国有资本、集体资本、非公有资本等交叉持股、融合发展。加快建立现代企业制度，对国有企业实行分类管理，扩大市属国有企业纳入国有资本经营预算管理范围。创新投融资体制机制。完善政策扶持体系，在城市基础设施建设、公益性项目建设等领域，加快推广PPP模式。鼓励采取吸纳社保基金和保险资金、引进境外资本等手段，降低融资成本。继续推进商事制度改革，强化后续市场监管。

推进财税体制改革。建立全面规范、公开透明的财政预算管理制度，完善政府预算体系，积极推进预决算公开。进一步推进依法治税工作，逐步建立财税优惠政策清单制度和公开制度，加大收费项目清理力度，严格非税收入缓减免，提升收入质量。出台财政拨款结余结转资金管理办法，完善预算编制与结余结转资金挂钩机制，进一步盘活财政沉淀资金。科学分类管理政府性债务，积极向上争取债券发行额度，严格控制政府性债务规模，落实债务清偿责任，防范和化解政府性债务风险。

推进“两型社会”综合配套改革。完成“两型社会”建设第二阶段改革。健全自然资源资产产权制度和用途管制制度，完善能源、资源节约集约使用制度，建立有效调节工业用地和居民用地合理比价机制。完善主体功能区制度，科学划定耕地、森林、湿地、水体等生态保护红线，强化国土空间开发保护。在韶山推进绿色GDP考核评价指标试点。开展国家公园体制改革试点，完善自然保护区、风景名胜区管理体制。建立生态环境损害责任终身追究制和环境污染事故追究制 飘邈之旅小说。推进环境污染第三方治理，推广第三方合同能源管理。建立资源有偿使用和生态补偿机制。

推进社会领域改革。深化教育领域综合改革。启动学区联盟办学改革。完善考试招生制度，推进中考制度改革。健全高中学生综合素质评价制度。启动中小学校长职级制和教师绩效工资分配改革试点，中小学校长、教师交流轮岗400名。加快现代职业教育改革，探索创建职业教育联盟，推动中高职衔接贯通工作。深入推进医药卫生体制改革，巩固和完善基本药物制度，全面推进县级公立医院改革，积极探索城市公立医院改革，加快基层卫生综合改革，努力构建“医联体”模式，支持社会办医。加快文化体制机制创新，深化公益性文化事业单位内部改革。

推进城乡一体化发展体制机制创新。推进土地管理制度改革。积极争取建设用地和补充耕地指标，保障经济社会发展用地需求。进一步加强土地市场调控，推进节约集约用地，加大对闲置土地、低效用地的处置力度，探索存量建设用地二次开发机制，依法依规解决土地遗留问题。探索建立不动产统一登记制度。开展集体经营性建设用地流转。加快推进落后小煤矿关闭退出。加快农村综合改革。全面推进农村土地承包经营权确权登记颁证。创新农业经营体制，培育国家级示范合作社5家、省级10家、市级20家，培育示范家庭农场20家。启动国有林场改革。积极稳妥推进农民宅基地、林权和住房财产权抵押、担保、转让。创新农村金融产品，继续拓展联户联保、订单担保和个人授信等贷款模式。加快实施城乡统一户口登记制度，启动居住证制度。

(三)推进新型城镇化，加快“北进中强”步伐。

加快“北进”，拓展发展空间。着眼长江经济带开放走廊建设，实施新型城镇化“三年行动计划”，聚集交通网络着力北进、兼顾东连，促进城市空间与产业空间契合。湘江风光带九华段和昭山段全线拉通。九华大道、昭山北路建成通车。推进长株潭城际铁路、益娄高速、京港澳高速殷家坳互通连接线建设。启用沪昆高速昭山南互通。启动G320湘潭绕城线城际快车道湘潭至湘乡段建设。加强与株洲连通，武广大道欧塘至G107段竣工通车，昭云路、铜板路加快建设。打造湘江黄金水道，启动湘潭易俗河港区一期、河西中心港综合水上服务中心建设。完善市域交通内网，强化内外通道互联互通，确保潭锰路响水段、福星东路等道路竣工通车，拉通宝塔南路、大湖南路等丁字路，加快一环东路建设，启动熙春北路、泗洲北路、望江西路等道路建设。加强桥梁管理和维护。完善城区慢行道路系统。湘潭公汽公司河西、河东新综合站场建成启用，湘潭客运中心站、韶山综合客运枢纽中心建设加快推进。建设多种运输方式无缝对接的综合性站场。

突出“中强”，提升城市承载能力。加速中心城市扩容提质，抓好“两房两棚”、“两供两治”工程。推进万楼新城、九华新城、岳塘新城建设。继续完善河东城区功能，加快河西城区更新改造。加快城市地下老旧管网改造，新城区逐步普及综合管廊。推进雨污分流，完善城区给水排水管网系统。加快河东第二污水处理厂及管网建设，河西、河东污水处理厂改扩建工程和湘潭经开区污水处理一期工程投入运行。加强垃圾处理设施、立体式停车场和地下停车场建设。深入推进工程建设质量治理“两年行动”。全面启动“智慧湘潭”建设。逐步提升城市管理精细化水平。以创建全国文明城市和国家卫生城市为抓手，继续强力推进超限超载、交通秩序、车窗抛物、噪音污染、工地扬尘等专项整治。清理整顿户外广告，加强夜景景观设计和建设，扮靓城市空间。大力加强居民小区管理。开展“微文明”市民行动。努力解决城区交通堵点问题。推进综合运输体系建设，大力发展“智能公交”。进一步规范出租车经营管理，核定出租车公司收费标准并向社会公示，严厉打击非法营运行为。加快城乡客运一体化建设，推动公共交通逐步向县(市)和中心城镇覆盖。在具备条件的村设立快递投送点。

加强生态建设，促进“两型”发展。落实省“一号重点工程”部署，深入推进湘江污染防治“三年行动计划”。规划和实施好竹埠港地区治理与开发。加快推进锰矿地区矿山地质环境治理示范工程二期、涉锰企业综合整治、历史固废治理和国家矿山公园建设。加强重金属污染耕地修复治理和种植业结构调整。加强农村面源污染治理，湘江干流两岸禁养区范围内规模养殖全部退出。全面推进大气污染防治行动，全力抗击雾霾。推进餐厨废弃物资源化利用和无害化处理国家试点城市建设。大力发展循环经济，工业固体废弃物综合利用率达到98%。推进国家新能源示范城市建设，严格执行建筑节能标准，推广可再生能源建筑应用120万平方米，完成建筑节能改造10万平方米。抓好城郊结合部、老旧小区、背街小巷等的绿化提质改造，实施公园、小游园增绿补绿工程，提升城市主次道路绿化水平，加快芙蓉大道等绿廊建设，启动宝塔公园、张家浸公园建设。推进爱劳渠综合整治。加强昭山“绿心”、水府庙水库等生态涵养区保护，加快水府庙国家湿地公园建设。开展国省道路两旁“裸露山地”绿化工作。完成营造林12.8万亩，森林覆盖率达到46.2%以上。

统筹城乡，推进一体化建设。加强农村公路、电力、供水、燃气、信息网络等基础设施建设。抓好农村公路大中修和管理养护。继续推进“万村千乡”市场工程和农贸市场标准化建设改造，完成50个村农网升级改造。加强农村水利基础设施建设，完成山坪塘清淤扩容3000口，建设高标准渠道300公里。加快国家重点镇和特色镇建设。完成特色镇专项规划、控制性详细规划和产业园区建设规划编制，完善特色镇路网、管网、污水和垃圾处理等基础设施。加强美丽乡村建设，科学编制村庄规划，完善基础设施，规范农村建房。启动新一轮农村环境卫生综合整治，重点抓好垃圾分类减量无害化处理和马路市场整治等工作。实施“点亮乡村”工程。继续抓好梅林桥城乡统筹示范片、姜畲现代农业示范区及30个美丽乡村等示范片区建设。加强移民地区、边远地区建设，重点推进65个省定贫困村和9个矿区村建设。

强化规划管控，提高建设效益。切实强化规划权威，坚决杜绝建设失序问题。高水平做好城市总体规划修改工作，进一步优化城市空间布局、功能布局和产业布局。推进宜居湘潭、特色湘潭规划，全面启动城市路网、地下管线普查和综合管网专项规划编制，完成宝塔路过江通道选址论证，完善河东城区等新城区民生设施规划，开展重要地段和区域城市设计。开展新型城镇化规划指导意见专题研究，加快编制完成村镇布局规划，做好特色农居点规划设计。提高规划的公众参与度。完善规划“一站式”服务和三维辅助审批系统建设。制定实施《城乡规划法》细则，加强规划实施的全方位、全过程监督，实行规划实施责任追究制度，确保规划严格落实。加大规划执法力度，完善常态化的联合执法机制，严厉查处违法违规建设。(四)切实保障和改善民生，维护社会和谐稳定。

实实在在改善民生。坚持以创业带就业，深入推进国家级创业型城市建设，鼓励自主创业，着重抓好高校毕业生、就业困难人员等重点群体就业，新增城镇就业5万人，新增农村劳动力转移就业1万人。切实维护农民工等就业人员合法权益。提高社会保障水平，推进全民社保登记试点，巩固城乡居民基本养老保险和基本医疗保险制度整合成果。实施机关事业单位养老保险制度改革。加大扶贫帮困力度，健全社会救助体系，建立常态化临时救助机制，设立市“救急难”公益基金，加大对突发意外家庭和特困家庭的救助力度。新增各类保障性住房和棚户区改造住房1万套，完成农村危房改造2000户。新建乡镇卫生院医务人员周转宿舍181套、农村学校教师周转宿舍748套。继续加强住房公积金归集扩面，加大对中低收入职工住房消费的支持力度。坚持稳销量、去存量、调结构、优品质、惠民生，促进房地产业持续稳定健康发展。

继续办好10件民生实事：提质改造农村村级组织活动场所100个；完善创新创业体系建设，其中扶持创业孵化基地10个，完成创业培训1万人；提质改造社区公园和老旧社区各5个；新增社会治安监控摄像头1000个；消除城市照明暗区802处；扶助全市60周岁以上失独老人890人左右，救治救助贫困重性精神病患者300名；贫困残疾人家庭无障碍改造300户；推进200个“帮扶村”建设，资助解决农村特困户建房200户；扶持蔬菜基地建设5个；建设城乡居民文化活动、体育健身场所100处。

提升公共服务水平。加快城乡教育优质均衡发展，新(改)建义务教育合格学校50所、公办幼儿园7所。完成教育信息化“三通两平台”建设，改善义务教育薄弱学校办学条件。调整优化市城区义务教育布局，加强城区学位建设。加快改善农村教师队伍结构，落实偏远地区教师津补贴发放制度。构建诚信医疗体系。加快市中心医院儿童诊疗中心、市三医院住院综合楼、市公共卫生服务中心等项目建设，建好8个乡卫生院、150个村卫生室。建立分级诊疗体系，试点基层首诊负责制。加快中医药事业发展。推进慢性病综合防控示范市创建。完善公共文化服务体系，组织引导城乡居民参加形式多样、有益身心健康的文体活动。大力规范文化市场，促进文化繁荣发展。完成市博物馆建设并开放运营，启动市图书馆、档案馆等城市文化工程建设。举办第四届中国(湘潭)齐白石国际文化艺术节。深化全民健身示范城市创建。大力发展社会养老服务，探索建立医养结合的养老服务新模式。深入实施妇女儿童发展规划，健全农村留守儿童、妇女关爱服务体系。坚持计划生育基本国策不动摇，大力推进“智慧人口”建设，抓好国家幸福家庭试点和国家婚育证明电子化改革试点。认真做好消防、侨务、外事、档案、史志、新闻出版、工青妇、供销、气象、水文、地震、水库移民、韶灌等工作。

全面加强社会治理。继续推进社区社会治理创新，加强社区社会管理服务平台建设，解决一批社区居委会无服务用房问题，开展农村社区建设试点，积极推进社区网格化管理和信息化建设，夯实基层基础。激发社会组织活力，鼓励和规范社会组织参与社会管理。继续推进市城区公安派出所建设。加快城乡电子治安防控体系和应急指挥中心建设。完善危机预防和管理体系，提高公共安全等的预防和应急处置能力。加强校园及周边环境综合治理，保障校园安全。强化安全保障能力建设，突出隐患排查和专项整治，用铁腕执法推动企业安全生产，严防安全事故发生。提高食品药品监管能力，推进肉菜流通追溯体系建设，保障饮食用药安全。加强市场综合监督管理，延伸消费维权网络，保障消费者合法权益。加强市场主体诚信体系建设，推进企业信用信息公示工作，促进信用信息的综合运用。推进金融生态城市创建，防范金融风险。健全社会矛盾纠纷预防化解机制，落实涉法涉诉信访依法终结机制，推行网上信访，引导群众依法逐级走访，进一步完善“市长热线”等平台，畅通民意表达渠道。加强社会治安综合治理，严密防范、依法打击各类违法犯罪行为，重点打击严重影响市民安全感的多发性犯罪、涉众型经济犯罪和阻扰项目建设等违法行为，维护社会和谐稳定，建设平安湘潭。

加强国防动员、民兵预备役和人防工作，推动军民融合深度发展。广泛开展全民国防教育和“双拥”共建活动，巩固军政军民团结局面。

编制全市国民经济和社会发展“十三五”规划，科学设计战略定位、发展导向和目标体系，描绘好未来发展蓝图。

三、推进法治政府和服务型政府建设，更好发挥政府作用

推进法治政府建设。认真落实中共湘潭市委关于全面推进依法治市、建设法治湘潭的实施方案。依法履行政府职能，坚持在宪法和法律范围内活动，提高运用法治思维和法治方式解决问题、推动工作的能力。坚持依法科学民主决策，完善政府议事决策规则，建立重大决策终身责任追究制及责任倒查机制，推行政府法律顾问制度。落实市政府常务会议学法制度。坚持法定职责必须为、法无授权不可为，严格按照法定权限和程序行权履责。推广相对集中执法，扩大委托乡镇执法试点，推行重点领域综合执法。规范行政执法行为，落实行政执法责任制，完善责令改正等纠错机制。强化对行政权力的制约和监督，自觉接受人大、政协和社会各界监督，完善行政裁决和行政复议制度，改进上级机关对下级机关的监督，对公共资金、国有资产、国有资源、领导干部经济责任履行情况实现审计监督全覆盖。全面推行政务公开，重点推进公共资源配置、重大建设项目批准和实施、社会公益事业建设等领域的政府信息公开，让权力在阳光下运行。

切实转变政府职能。完成政府机构改革，精简、规范机构设置，优化职能配置，提高政府效能。推进政事分开、事企分开和管办分离，加快事业单位分类改革。加大政府购买公共服务力度，制定政府购买公共服务目录。大力推进简政放权，继续做好行政审批事项的承接落实和取消下放工作，消除行政审批“灰色地带”，整治“红顶中介”，全面推行负面清单制度并实行动态管理，探索对负面清单外符合市场准入标准的项目实行零审批。建立健全科学合理的监管制度，强化监管职能。坚持“两集中、两到位”，严格落实“一门受理”、“一站办结”等制度，进一步优化审批流程、压缩审批时限，推行并联审批，提高政务服务中心运行效率。加快电子政务平台建设，采用手机客户端、自助服务终端等方式，建设全市统一的网上政务平台，推动审批服务事项网上办理。改善基层工作人员工作和生活条件，稳定基层工作队伍，切实增强基层运转保障能力。

持续加强作风建设。严格落实中央、省委、市委加强作风建设各项规定，巩固党的群众路线教育实践活动成果，自觉践行“三严三实”，始终做到心中有党、心中有民、心中有责、心中有戒。要坚定正确的政治方向，坚决维护中央权威，在重大问题上立场坚定，不论什么时候，遇到什么情况，都经得起风浪考验，做到令行禁止。要恪守为民之责，心系人民群众，从群众愿望出发定任务、抓工作，尽心尽力把群众的事情办好，特别是要多关心困难群众，多做雪中送炭的事情。要强化责任担当，弘扬实干精神，保持满腔热情，对确定了的目标任务，起而行之，锲而不舍，直至成功。要严守政治纪律和政治规矩，做到心有所畏、言有所戒、行有所止，全面落实廉洁从政各项规定，加快廉政风险防控体系建设，坚决纠正“四风”，反对腐败。继续执行政府工作人员“约法三章”，完善“三公”经费、楼堂馆所、办公用房等各项管理制度，推行公务卡结算制度，节约政府开支，降低行政成本。政府各级干部要严格自律，带头提升道德境界，强化职业操守，正确处理公和私、情和法、利和法的关系，恪守诚信，维护公平正义，凝聚和传播正能量。加强行政监察，严格行政追责。对政府工作人员贪腐行为坚持“零容忍”，发现一起，查处一起，绝不姑息!坚持以政风带民风，营造风清气正的经济社会发展环境。

各位代表，今年形势依然十分复杂严峻，发展改革的任务同样非常繁重。让我们在中共湘潭市委的坚强领导下，开拓创新，负重奋进，坚决完成今年经济社会发展和“十二五”规划的各项目标任务，为更好开启“十三五”工作、提前全面建成小康社会、加快幸福湘潭进程而努力奋斗!
